# Supplementary material for: Benefits and Pitfalls of HPLC Coupled to Diode-Array, Charged Aerosol, and Coulometric Detections: Effect of Detection on Screening of Bioactive Compounds in Apples
Source: Molecules. 2021 May 28;26(11):3246. doi: 10.3390/molecules26113246 (PMC8199029; doi:10.3390/molecules26113246)
Supplement: Supplementary file 1 [file molecules-26-03246-s001.zip › molecules-1208483-supplementary.pdf]

Supplementary material

Benefits and pitfalls of HPLC coupled to diode-array, charged aerosol, and coulometric detections: Effect of detection on screening of bioactive compounds in apples

Marcela Hollá [1], Aneta Bílková [1,2], Pavel Jakubec [1], Stanislava Košková [1], Hana Kočová Vlčková [1], Dalibor Šatínský [1], František Švec [1], Hana Sklenářová [1*]

[1] Department of Analytical Chemistry, Faculty of Pharmacy in Hradec Králové, Charles University, 50005 Hradec Králové, Czech Republic

[2] Research and Breeding Institute of Pomology Holovousy Ltd., 50801 Hořice, Czech Republic;

* Correspondence: sklenarova@faf.cuni.cz; Tel.: +420-495-067-453 (H.S.)

LC-HRMS characterization of quantified compounds in apple samples

Acquity Ultra Performance LC™ (UPLC) I-Class system (Waters, Milford, MA, USA) coupled with Synapt-G2-Si quadrupole-time-of-flight (Q-TOF) mass spectrometer (Waters, Milford, MA, USA) was used for characterization of phenolic compounds present in apple samples. Separation using Acquity BEH Shield RP18 analytical column (100 mm x 2.1 mm; 1.7 µm) with mobile phases 0.1% formic acid in water (eluent A) and MeOH (eluent B) according to the following gradient program 5%B 2 min, 95% in 11 min, 95%B 2 min, 5%B in 0.25 min, and 1.75 min equilibration at a flow rate 0.3 mL/min. Injection volume 5 µL was used. The total time of chromatographic separation, including column equilibration, was 17 minutes.

For detection, Q-TOF with electrospray ionization in negative mode (ESI^-) was used. The optimal set up of ion source, ion optic and analyzer parameters were followings: capillary voltage -1.5 kV, sampling cone: 5 V, source offset: 40 V, ion source temperature 120 °C. The desolvation gas (nitrogen) flow 800 L/hr, and temperature 600 °C was used. Nitrogen was also used as a cone gas with flow rate 50 L/hr and nebulization gas with pressure 3.5 bar. Argon was used as the collision gas.

MS scans acquired in the range of *m/z* 50 – 1200 and MS/MS scans of individual compounds in negative ionization mode were used for confirmation of compounds. Leucine encephalin at a concentration 200 pg/µL was used as an internal calibrant and 0.5 mM sodium formate solution as an external calibrant. The MassLynx 4.1 Data System was used for system control, data acquisition, and data evaluation.

Table S1. Results of cultivars' phenolic profiles determined by DAD and CAD expressed as single determined phenolic compound ± SD and total quantified phenolics in µg/g ± SD (n=3). Separation using the Luna Omega Polar C18 (150 mm x 4.6 mm; 5.0 µm) fully porous column with mobile phases aqueous acetic acid with pH 2.8 (A) and acetonitrile (B) according to the following gradient program 10-50% B in 10 min, 50% B 0.2 min, 50-10% B in 2.3 min at a flow rate 1.0 mL/min. The temperature of the column was held at 30°C, and injection volume 10 µL was used.

Cultivar/ analyte	Chlorogenic acid		Epicatechin		Rutin		Phloridzin		Quercetin		TOTAL	TOTAL
	DAD 320 nm	CAD	DAD 280 nm	CAD	DAD 365 nm	CAD	DAD 280 nm	CAD	DAD 365 nm	CAD	DAD	CAD
'Angold'	33.51 ± 0.05	33.75 ± 1.04	<LOQ	<LOQ	4.28 ± 0.01	8.24 ± 0.31	1.41 ± 0.01	<LOQ	<LOQ	<LOQ	39.20 ± 0.16	41.99 ± 1.58
'Artiga'	46.13 ± 1.43	46.29 ± 2.29	7.12 ± 0.53	14.16 ± 0.77	16.36 ± 0.65	17.69 ± 0.67	3.75 ± 0.07	<LOQ	<LOQ	<LOQ	73.36 ± 5.49	78.14 ± 4.24
'Benet'	13.99 ± 1.65	13.84 ± 2.60	<LOQ	11.50 ± 1.64	2.15 ± 0.09	11.65 ± 0.76	5.71 ± 0.42	13.45 ± 0.97	<LOQ	<LOQ	21.85 ± 2.58	50.43 ± 9.46
'Golden Delicious'	8.80 ± 1.25	9.58 ± 1.89	<LOQ	4.61 ± 0.28	<LOQ	<LOQ	2.88 ± 0.42	<LOQ	0.72 ± 0.00	<LOQ	12.40 ± 1.80	14.19 ± 2.79
'Golida'	1.74 ± 0.10	<LOQ	6.36 ± 0.11	16.87 ± 0.78	10.83 ± 0.11	14.35 ± 1.00	2.31 ± 0.13	11.91 ± 0.79	1.09 ± 0.01	<LOQ	22.33 ± 1.29	43.14 ± 3.01
'Jarka'	13.51 ± 1.29	14.82 ± 1.22	1.49 ± 0.16	12.15 ± 0.44	3.08 ± 0.28	8.86 ± 0.26	2.61 ± 0.25	9.33 ± 0.32	1.11 ± 0.02	<LOQ	21.81 ± 2.29	45.17 ± 3.71
'Lady Silvia'	12.30 ± 2.38	11.47 ± 2.50	10.03 ± 1.90	7.44 ± 0.79	<LOQ	<LOQ	2.97 ± 0.50	<LOQ	1.10 ± 0.01	<LOQ	26.40 ± 5.11	18.91 ± 4.11
'Melrose'	10.28 ± 0.80	10.78 ± 0.52	1.19 ± 0.27	9.24 ± 1.00	1.25 ± 0.04	<LOQ	4.42 ± 0.49	4.88 ± 0.65	1.08 ± 0.01	<LOQ	18.22 ± 4.08	24.90 ± 3.30
'Meteor'	2.23 ± 0.16	<LOQ	<LOQ	7.30 ± 0.47	2.88 ± 0.12	<LOQ	1.58 ± 0.45	<LOQ	<LOQ	<LOQ	6.69 ± 1.91	7.30 ± 0.47
'Red Jonaprince'	8.86 ± 0.33	9.60 ± 0.51	<LOQ	<LOQ	<LOQ	<LOQ	2.17 ± 0.07	4.94 ± 0.29	<LOQ	<LOQ	11.03 ± 0.42	14.53 ±0.85
'Reluga'	3.39 ± 0.87	<LOQ	13.36 ± 3.20	17.99 ± 3.02	4.82 ± 0.18	<LOQ	3.08 ± 0.61	<LOQ	1.10 ± 0.03	<LOQ	25.75 ± 6.59	17.99 ± 3.02
'Resista'	10.98 ± 1.87	10.55 ± 1.80	2.32 ± 0.38	33.66 ± 1.03	9.90 ± 0.32	24.48 ± 0.49	5.73 ± 0.90	6.22 ± 0.69	1.14 ± 0.02	<LOQ	30.07 ± 5.11	74.91 ± 12.78
'Rubinola'	68.73 ± 2.27	69.51 ± 3.13	19.17 ± 0.67	18.78 ± 1.79	<LOQ	<LOQ	7.57 ± 0.26	7.73 ± 0.49	<LOQ	<LOQ	95.47 ± 3.32	96.02 ± 9.13
'Rubinstep'	5.88 ± 1.08	<LOQ	3.07 ± 0.56	<LOQ	1.35 ± 0.03	<LOQ	1.93 ± 0.34	5.63 ± 0.37	1.08 ± 0.01	<LOQ	13.30 ± 2.45	5.63 ± 0.37
'Santana'	19.37 ± 0.18	18.95 ± 1.39	<LOQ	<LOQ	2.71 ± 0.12	<LOQ	3.34 ± 0.19	<LOQ	1.08 ± 0.01	<LOQ	26.50 ± 1.51	18.95 ± 1.39
'Topaz'	5.26 ± 1.29	<LOQ	3.58 ± 0.92	7.22 ± 0.67	3.27 ± 0.06	<LOQ	0.99 ± 0.14	<LOQ	<LOQ	<LOQ	13.10 ± 3.36	7.22 ± 0.67

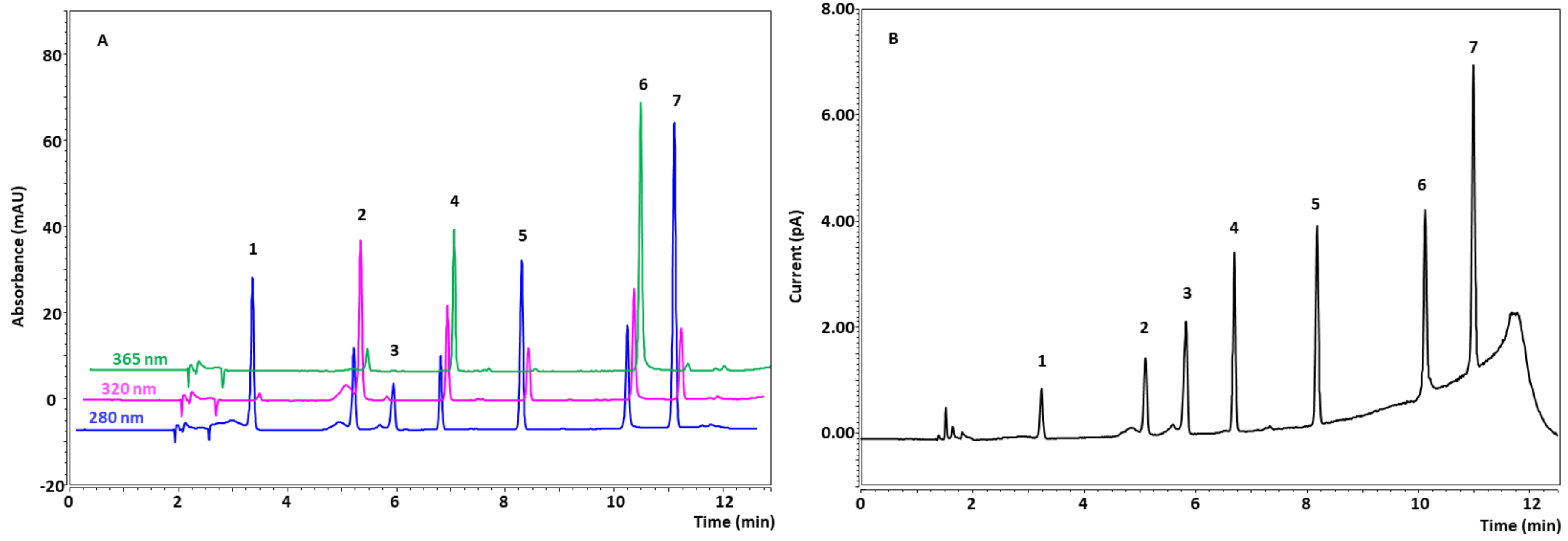

Figure S1. Chromatogram of mixed standard solution (10 µg/mL) separation using the Luna Omega Polar C18 (150 mm x 4.6 mm; 5.0 µm) fully porous column with mobile phases aqueous acetic acid with pH 2.8 (A) and acetonitrile (B) according to the following gradient program 10-50% B in 10 min, 50% B 0.2 min, 50-10% B in 2.3 min at a flow rate 1.0 mL/min. The temperature of the column was held at 30°C, and injection volume 10 µL was used. Acquired by diode array detector (A, signal and time offset) and charged aerosol detector (B). Peaks: gallic acid 1, chlorogenic acid 2, epicatechin 3, rutin 4, phloridzin 5, quercetin 6, phloretin 7.

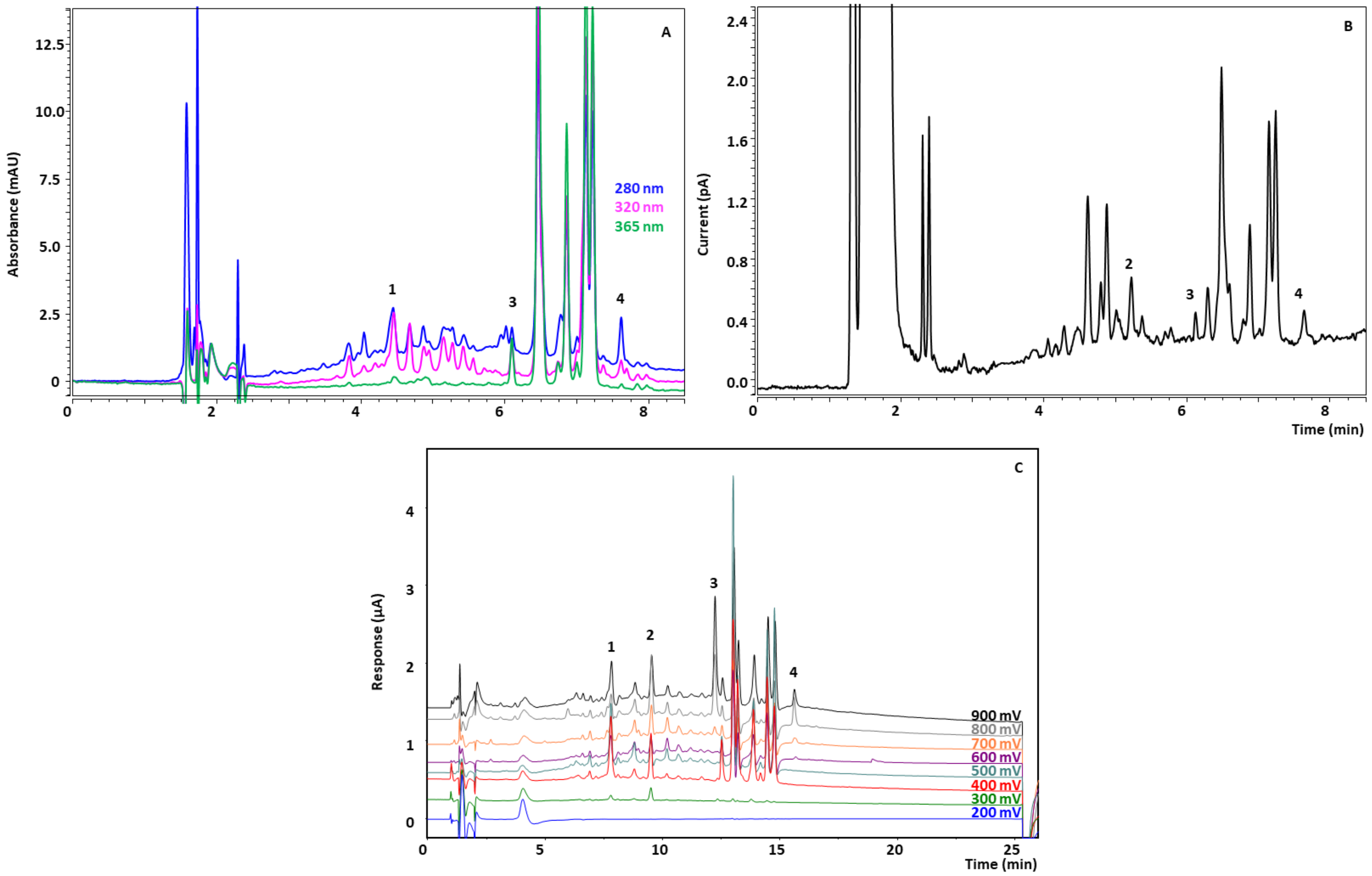

Figure S2. Analysis of 'Meteor' apple extract acquired on diode array (A), charged aerosol (B) and coulometric detector (C). Separation using the Luna Omega Polar C18 (150 x 4.6 mm; 5.0 µm) fully porous column with mobile phases aqueous acetic acid with pH 2.8 (A) and acetonitrile (B) according to the following gradient program 10-50% B in 10 min, 50% B 0.2 min, 50-10% B in 2.3 min at a flow rate 1.0 mL/min. The temperature of the column was held at 30°C, and injection volume 10 µL was used. Peaks: chlorogenic acid (1), epicatechin (2), rutin (3), and phloridzin (4).

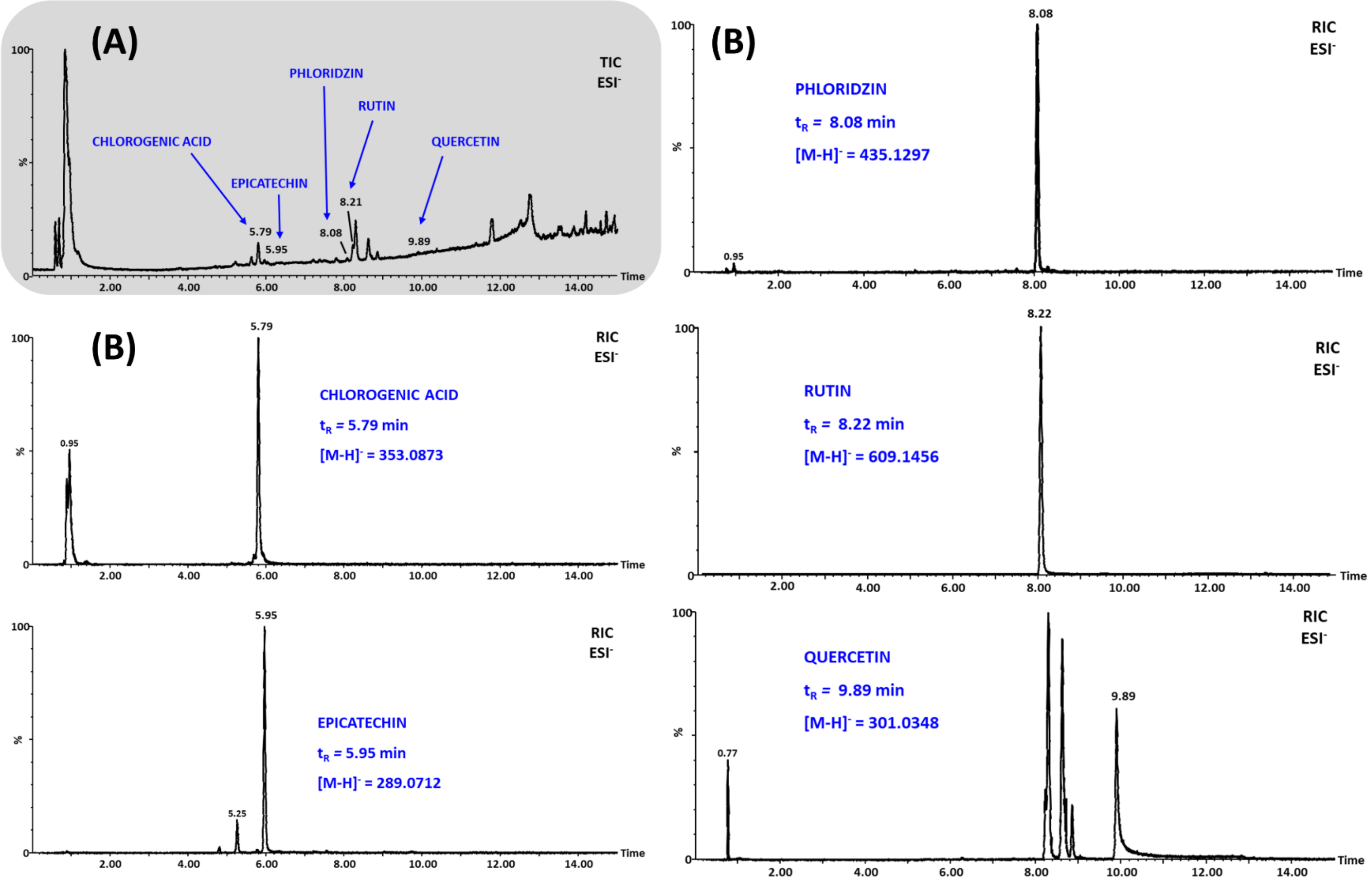

Figure S3. Chromatograms of apple extract sample obtained in negative ionization mode: (A) total ion chromatogram (TIC) of apple extract, (B) reconstructed ion chromatograms (RIC) of identified compounds (chlorogenic acid, epicatechin, phloridzin, rutin and quercetin) in apple extract.

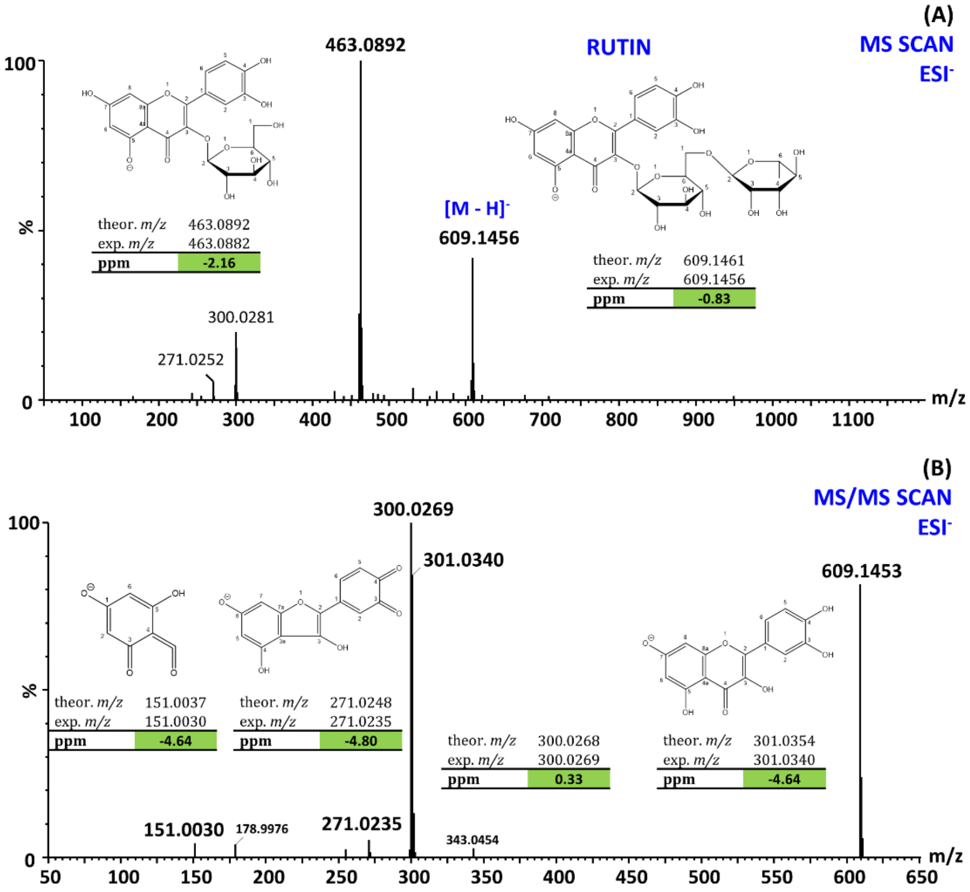

Figure S4. (A) MS scan of rutin and (B) MS/MS scan of deprotonated molecule with *m/z* 609.1461. Both spectra were obtained from measurement of apple extract sample in negative ionization mode.

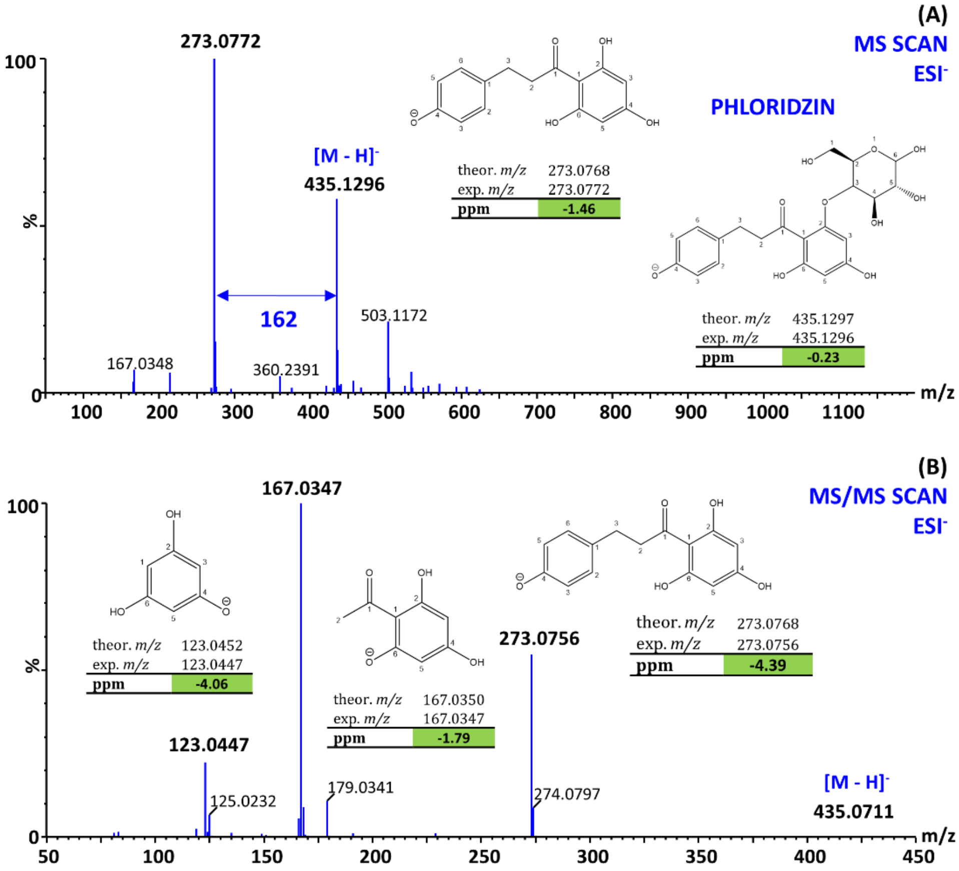

Figure S5. (A) MS scan of phloridzin and (B) MS/MS scan of deprotonated molecule with *m/z* 435.1297. Both spectra were obtained from measurement of apple extract sample in negative ionization mode.

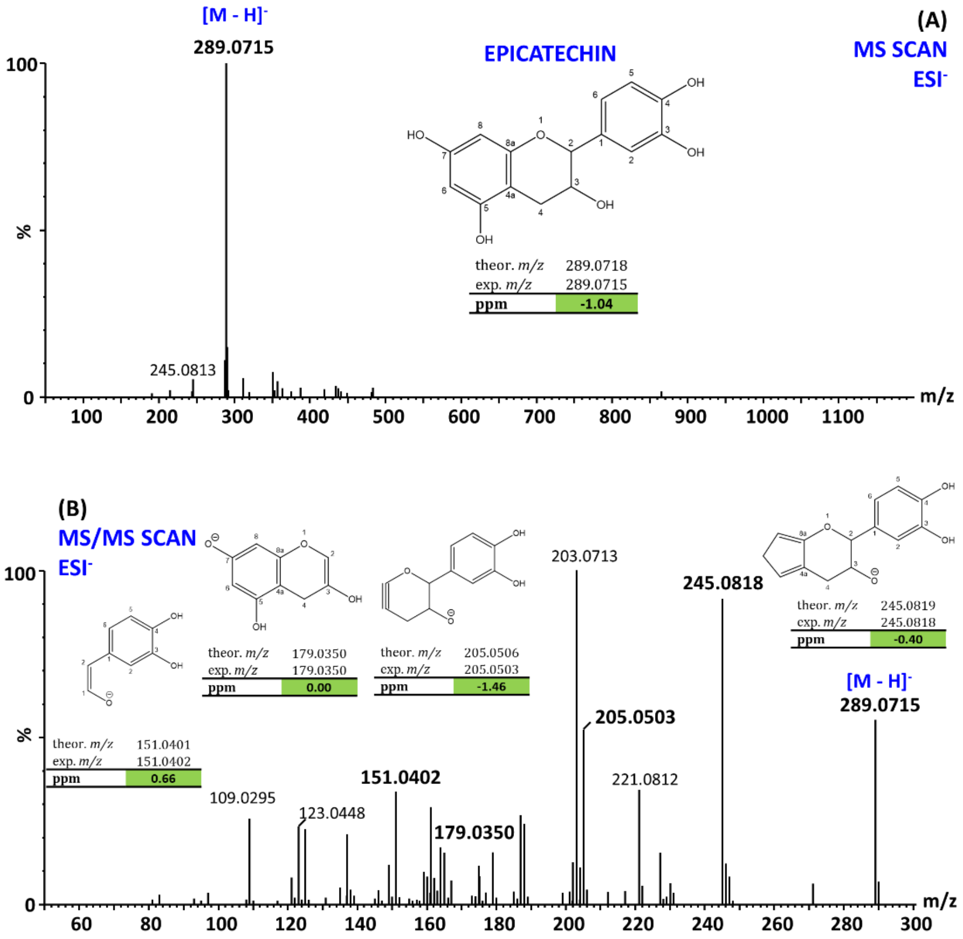

Figure S6. (A) MS scan of epicatechin and (B) MS/MS scan of deprotonated molecule with *m/z* 289.0718. Both spectra were obtained from measurement of apple extract sample in negative ionization mode.

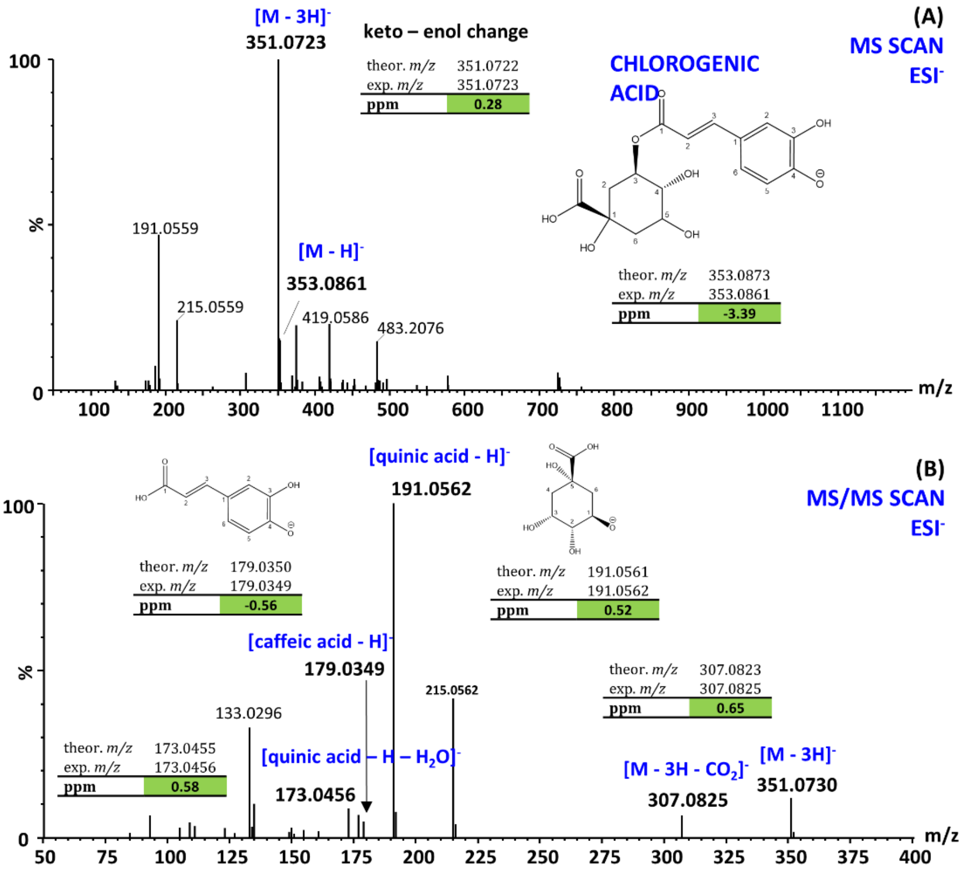

Figure S7. (A) MS scan of chlorogenic acid and (B) MS/MS scan of ion with *m/z* 351.0722. Both spectra were obtained from measurement of apple extract sample in negative ionization mode.

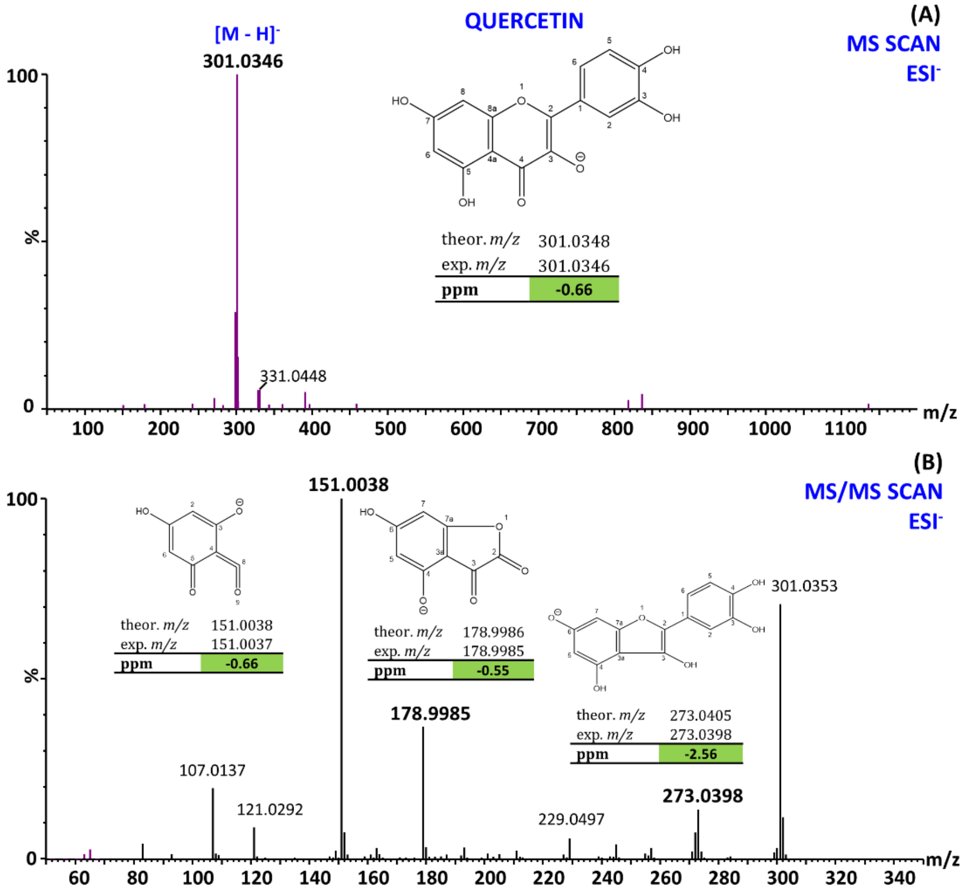

Figure S8. (A) MS scan of quercetin and (B) MS/MS scan of deprotonated molecule with *m/z* 301.0348. Both spectra were obtained from measurement of apple extract sample in negative ionization mode.
